# Need for Cognition Among Users of Self-Monitoring Systems for Physical Activity: Survey Study

**DOI:** 10.2196/23968

**Published:** 2021-10-14

**Authors:** Kirsi Halttu, Harri Oinas-Kukkonen

**Affiliations:** 1 Oulu Advanced Research on Service and Information Systems Research Unit Faculty of Information Technology and Electrical Engineering University of Oulu Oulu Finland

**Keywords:** self-monitoring, wearables, physical activity tracking, mHealth, need for cognition, persuasive design, tailoring, user research, mobile phone

## Abstract

**Background:**

Need for cognition (NFC) is among the most studied personality traits in psychology. Despite its apparent relevance for engaging with technology and the use of information, it has not been studied in the context of self-monitoring systems and wearables for health. This study is the first to explore the relationship between NFC and commercial self-monitoring systems among healthy users.

**Objective:**

This study aims to explore the effect of NFC levels on the selection of self-monitoring systems and evaluation of system features of self-monitoring and feedback, as well as perceived credibility and perceived persuasiveness. We also assessed perceived behavior change in the form of self-reported activity after adopting the system.

**Methods:**

Survey data were collected in October 2019 among university students and personnel. The invitation to respond to the questionnaire was addressed to those who had used a digital system to monitor their physical activity for at least two months. The web-based questionnaire comprised the following 3 parts: details of system use, partially randomly ordered theoretical measurement items, and user demographics. The data were analyzed using structural equation modeling. The effect of NFC was assessed both as 3 groups (low, moderate, and high) and as a continuous moderator variable.

**Results:**

In all, 238 valid responses to the questionnaire were obtained. Individuals with high NFC reported all tested system features with statistically significantly higher scores. The NFC also had some effect on system selection. Hypothesized relationships with perceived credibility gained support in a different way for individuals with low and high NFC; for those with low NFC, credibility increased the persuasiveness of the system, but this effect was absent among individuals with high NFC. For users with high NFC, credibility was related to feedback and self-monitoring and perhaps continuously evaluated during prolonged use instead of being a static system property. Furthermore, the relationship between perceived persuasiveness and self-reported activity after adopting the system had a large effect size (Cohen f^2^=0.355) for individuals with high NFC, a small effect size for individuals with moderate NFC (Cohen f^2^=0.107), and a nonsignificant path (*P*=.16) for those with low NFC. We also detected a moderating effect of NFC in two paths on perceived persuasiveness but only among women. Our research model explained 59.2%, 63.9%, and 47.3% of the variance in perceived persuasiveness of the system among individuals with low, moderate, and high NFC, respectively.

**Conclusions:**

The system choices of individuals seem to reflect their intrinsic motivations to engage with rich data, and commercial systems might themselves be a tailoring strategy. Important characteristics of the system, such as perceived credibility, have different roles depending on the NFC levels. Our data demonstrate that NFC as a trait that differentiates information processing has several implications for the selection, design, and tailoring of self-monitoring systems.

## Introduction

### Theoretical Background

Monitoring oneself in terms of behavior is one of three subfunctions in self-regulation models [1] and is elementary to behavior change. Self-monitoring is a behavior change technique (BCT) that refers to keeping a record of specified behavior related to a target behavior change domain or outcome of behaviors, such as weight loss [2]. Increasing numbers of individuals are turning to their smartphones and additional sensing devices such as wearables to collect data on their own behavior and to facilitate their personal self-regulation needs. Self-monitoring improves self-awareness; increases self-knowledge; and makes habitual, often unconscious, behavioral patterns more visible. Although many benefits of self-monitoring can also be attained with nondigital approaches, technology has increased its potential [3,4]. Currently, self-monitoring is the most common BCT among mobile apps designed to support physical activity [5] and health behavior change [6] and in health interventions [7], and it is the core function of activity trackers [8,9].

BCTs derived from self-regulation theory [10] and control theory [11,12] are generally considered effective. Self-monitoring combined with other elements of self-regulation, such as prompting intention formation and goal setting, providing feedback, and reviewing goals, is considered the most effective technique for achieving more physical activity and healthier eating [13]. Many self-monitoring systems have implemented theory- and evidence-based techniques [14] and present a viable and useful approach to support self-regulation toward healthier behaviors [15].

Self-monitoring is also a persuasive feature because of its ability to influence thinking and is part of the persuasive systems design (PSD) model [16]. Self-monitoring systems are, indeed, persuasive systems implementing persuasion, “a deliberate attempt to change attitudes or behaviors or both” [17]. A general theory of attitude change, the elaboration likelihood model (ELM) [18,19], is a cognitively oriented model of persuasion that explains how an influence process may affect attitude and behavior change. The ELM is based on the concept of message elaboration through central or peripheral routes that represent different levels and types of information processing: when presented with a message, individuals may process information through careful consideration (central route) or more automatically (peripheral route), relying on effortful analysis instead of simple decision rules [20]. Any piece of information, regardless of whether it is a rigorously designed motivational message or a small detail in the implementation of software or the environment of the persuasion event, can change the elaboration mode in a situation. This *multiple roles* notion suggests that situational factors also affect the likelihood and extent of elaboration, and any variable can influence it by serving as an argument, cue, determinant of the extent of elaboration itself, or source of bias [21,22].

The personal relevance of the argument is one of the strongest variables exerting an effect on the motivation to elaborate. Petty and Cacioppo [23] regard this construct as the personal meaning and intrinsic importance of an issue that people expect “to have significant consequences for their own lives” [24]. Behavior change approaches that aim to increase the personal relevance of information provided, thereby creating a more optimal environment for persuasion [25,26], are tailoring and personalization strategies. The effectiveness of these strategies is presumably based on increased involvement in, and engagement with, the subject matter [27], both possible outcomes of increased personal relevance [28]. Tailoring might use any part of the system, but most often, informational content is tailored to contain more relevant information for particular groups of users. To summarize, tailoring improves the fit between the user and the system, and it usually focuses on motivation to elaborate the information provided.

Tailoring is based on the assumption that target audiences differ in terms of the selected tailoring trait. There are rather stable individual differences in the intrinsic motivation to engage in extensive thinking and enjoy effortful cognitive activities, such as the need for cognition (NFC) [29]. The implications of the extensive studies on this personality trait are that individuals with high NFC have stronger information-seeking habits [30] and that they are in general more influenced by argument quality than peripheral cues [31]. They are also more motivated to process messages that they perceive as complex [32] and are more easily persuaded using cognition-based messages [33]. There are some preliminary indications that NFC affects how individuals interact with, and use, software, which implies that information processing and NFC itself affect both behaviors and actions. For example, those with high NFC use adaptive user interface features more frequently [34] and prefer personalized content and choose more preference-matched offers compared with individuals with low NFC [35]. These examples from existing studies support the relevance of NFC to interactive systems such as self-monitoring systems that provide considerable amounts of information to process.

One might expect that individuals who adopt and continue to use self-monitoring systems are inherently interested in information and are able to base their attitudes and behaviors accordingly. However, research has not addressed these assumptions, and it is not known how NFC is associated with the selection of systems or the evaluation of system features. In this study, we aim to fill this gap by exploring how individual differences in NFC influence the evaluations of self-monitoring system features in commercial systems for physical activity. Our survey sample comprised individuals who use these systems volitionally and have selected their systems themselves. In this setting, we examine whether users with different levels of NFC select their systems similarly or evaluate self-monitoring features differently and whether this information is relevant for future tailoring approaches.

### Model Development and Hypotheses

#### Research Model

**Figure 1:**
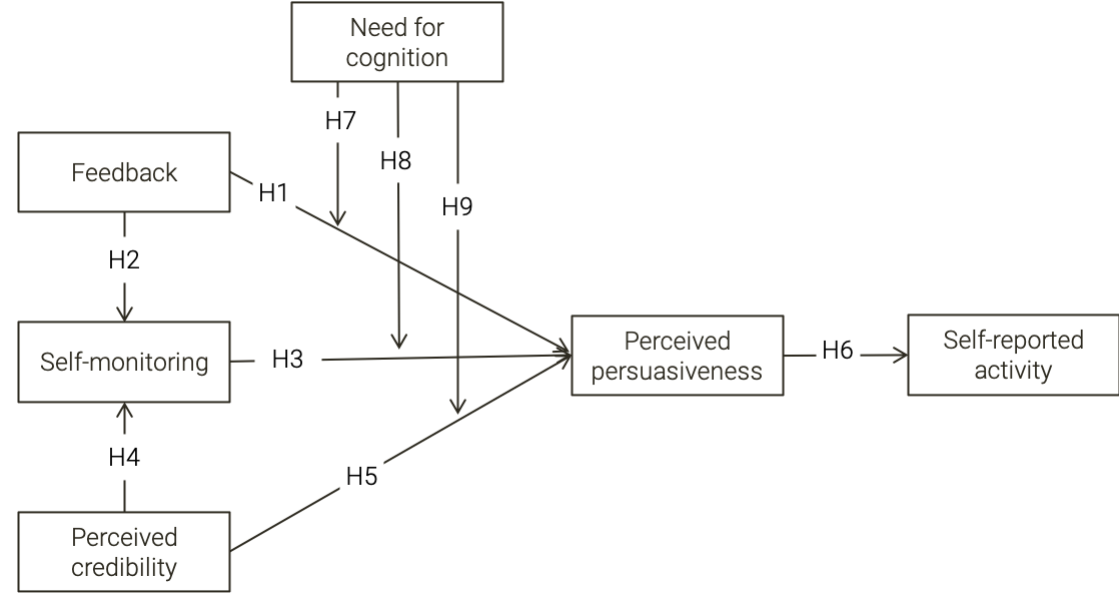
Research model and hypotheses.

We built a research model on the relationships among elements based on the PSD model [16]. These relationships have been validated in several studies using both web-based and mobile apps. All the relationships among the constructs, represented by the arrows (Figure 1), are assumed to be positive.

#### Feedback and Perceived Persuasiveness

Feedback on behavior or on outcomes of behaviors can be considered *good* if it is delivered at the right time, is personally relevant, and is actionable [36]. On the basis of these assumptions, our feedback construct was created to measure these general functional qualities of feedback features rather than the actual content in terms of argument quality or wording. The connection between elaboration and persuasion refers to any change that results from exposure to communication [18], that is, the feedback the system provides to the user. Feedback features, regardless of being status information with numbers or words or instructions on how to reach the goal, form the arguments for persuasive attempts. Therefore, we expect the following relationship between feedback and subjective persuasiveness evaluation:

H1: Feedback functionality is positively related to the perceived persuasiveness of the system.

#### Feedback and Self-Monitoring

Feedback is an essential part of self-monitoring activity. It provides information for both setting realistic goals and evaluating goal attainment [10]. Feedback features can be implemented in many forms, and actual feedback can be provided through numbers, messages, and graphics. Some wearables also have sound, vibration, or lights that communicate the user’s current status. Feedback is often obtained by taking quick glances at a device [37], but some devices do not have screens at all and provide feedback only through a mobile app (eg, basic pedometers, some activity wristbands, and smart rings). In any case, looking at data does not necessarily mean that they have been understood and reflected on [38]. According to the study by Michie et al [2], feedback and self-monitoring are categorized as different techniques, and both can target either behaviors or outcomes of behaviors. We followed this approach by developing our own construct for both techniques but aimed to keep them as general principles without specifically focusing on behaviors or their outcomes. This approach enables the evaluation of several systems by using the same constructs and is also congruent with system design practice: both self-monitoring and feedback can be designed and implemented in many different ways, and either might be dysfunctional in terms of the persuasiveness and behavior change support of the system.

Self-monitoring combined with feedback or other self-regulation– or control-theory–driven techniques such as giving feedback is considered effective for physical activity support [13]. The positive relationship between dialog support and the primary task features of the PSD model—feedback and self-monitoring features developed from these constructs of the model—has also been reported elsewhere [38-41]. We set the following hypothesis to evaluate the theorized and previously reported relationship between feedback and self-monitoring:

H2: Feedback functionality is positively related to self-monitoring features.

#### Self-Monitoring and Perceived Persuasiveness

Self-monitoring is a BCT where an individual monitors and records their own behavior (eg, number of steps per day) or outcomes of behaviors (eg, weight; Michie et al [2]). Self-monitoring is a functionality that has been shown to affect continuance intention through its perceived usefulness [15] and potential to disrupt unwanted habits [42,43]. Its ability to evoke self-adjustment actions when information about the monitored factor is presented has its roots in a phenomenon known as reactivity, referring to changes in behavior that occur because of merely being monitored by someone else. The reactivity effects of monitoring seem to be similar when a person is self-monitoring [44]. However, self-monitoring activity itself is not perceived as strongly motivating in cases where users have not chosen to start it based on personal motivations [45]. In addition, the notoriously high abandonment rates of self-monitoring tools imply that mere self-monitoring does not always increase the persuasiveness or desirability of a device. These findings imply that the persuasive nature and reactivity of self-monitoring might be based on certain pre-existing motivations to understand oneself or on behavior-change goals [46] or that the practicalities of actual self-tracking are often too difficult to maintain in the long term.

However, self-monitoring is often referred to as a persuasive feature [17,47] and is included as one of the primary task support features in the PSD model [16]. Previous research has shown that primary task support features increase the perceived persuasiveness of a system [39,40]. Because of the personal relevance of the self-monitoring data and self-selected nature of continued use of the system, we expect that self-monitoring features do influence the persuasiveness of the system. Hence, we set the following hypothesis:

H3: Self-monitoring is positively related to the user’s perceived persuasiveness evaluation.

#### Perceived Credibility and Self-Monitoring

The credibility of the system is the core of the process. Users must trust the system to provide accurate information in terms of progressing toward, and reaching, their goals, as well as understanding their current status. High credibility is mandatory for a system that instructs or advises its users, reports measurements, and provides information and analysis. All these characteristics are typical of self-monitoring systems. If users trust the system, they are more likely to consider the advice provided and follow the recommendations. Therefore, the act of self-monitoring is highly dependent on the perceived credibility of the system. Alternatively, the system might be abandoned. The perceived credibility construct refers to trustworthiness, believability, and reliability, all relevant for the subjective evaluation of credibility. Therefore, we hypothesize as follows:

H4: The perceived credibility of the system has a positive influence on self-monitoring features.

#### Perceived Credibility and Perceived Persuasiveness

In persuasive communication, source credibility refers to the effects of the credibility of the message source. The nature of source credibility is perceived, that is, it refers to a subjective evaluation of several source-related context variables that have been shown to alter persuasion outcomes, for example, in child obesity campaigns [48]. However, the literature usually denotes source credibility as comprising expertise and trustworthiness [49-52]. A credible source is usually more persuasive and influential and is evaluated more favorably [53]. Credibility refers to an object of interest (the system in this case) and the trust of the message recipient: whether (or not) the recipient trusts the system [54]. The trustworthiness and precision of the information provided by the device are important in terms of its effectiveness and overall evaluations of the system [55]. Although trust in a service provider is a somewhat larger phenomenon in the context of self-monitoring systems that often entail both device and software, the findings in the field of health information systems indicate that credibility forms through interaction with the system or service [56] and transforms into trust with prolonged use [57]. Issues that might endanger the credibility of the system include poor style and errors [54], which signal a lack of expertise and a decrease in trust.

Our construct queries the trustworthiness of the system, believability of the measurements, and expertise as impressions that the system was built by professionals. The relationship between perceived credibility and perceived persuasiveness has previously been reported as significant and positive [39,40] in terms of the PSD model constructs that we have used as the baseline for our items. On the basis of existing studies on how the credibility of systems is developed and how self-monitoring systems should perform, we offer the following hypothesis:

H5: Perceived credibility has a positive relationship with perceived persuasiveness.

#### Perceived Persuasiveness and Subjective Activity Change

The study by Lehto [58] defines perceived persuasiveness as “individuals’ favorable impressions toward the [behavior change support systems].” Therefore, it has an attitudinal component. The construct is created to measure subjective rather than objective persuasiveness, which is a variable that requires measuring actual behavior or attitude changes. The measurement items of the construct encourage thinking about the system’s influence, for example, by inviting metacognitive processing, which has been connected to high message elaboration conditions [59]. Previous studies have reported that the perceived persuasiveness of a system has a positive influence on continuance intention, intention to use, and intention to adopt health behavior change support systems [39,40]. We hypothesize that the salience of these self-evaluative thoughts positively influences self-reported activity:

H6: Perceived persuasiveness has a positive influence on self-reported level of activity after adopting a self-monitoring system.

#### NFC as Moderator

##### Personal Relevance of Overall Feedback

In the context of the volitional use of self-selected systems used for self-monitoring, we have assumed that the personal relevance of overall feedback is rather high. The data collected are always personal behavioral data, and the insights gained through the information that the data provide are similar to personalized or tailored messages because they are based on the user’s own measurement data. However, not all information is of the same relevance, depending on an individual’s goals, current situation, or more long-standing lifestyles.

##### Feedback and Perceived Persuasiveness

The feedback construct was created to measure the general functional qualities of feedback features instead of their actual content in terms of argument quality or wording. Previously, quantified types of motivation to use self-monitoring systems had been connected to both affective and informational feedback [60]. In the context of wearable trackers and smartphone apps with sensors, we consider the content of the feedback to be cognitive in nature as well. Cognition-based messages have been identified as more persuasive and attention-enhancing among individuals with high NFC than among individuals who have a high need for affect [33] and individuals with low NFC. This effect has also recently been demonstrated using neuroscientific methods [61]. Feedback can also be categorized as an assessment type of information, which has recently been linked to individuals with high NFC [62]. Therefore, we expect that NFC moderates the relationship between feedback and perceived persuasiveness, leading to the following hypothesis:

H7: NFC moderates the relationship between feedback and perceived persuasiveness. The positive influence of feedback is stronger for individuals with high NFC because of the informative nature of feedback in self-monitoring systems.

##### Self-Monitoring and Perceived Persuasiveness

The tendency demonstrated by NFC is heavily geared toward cognitive functions. NFC correlates positively with objectivity and refers to a tendency to rely on empirical information and rational consideration [63]. Messages based on cognition were more persuasive than affective messages among individuals with high NFC compared with individuals with low NFC; NFC also influenced receptivity to cognition-based messages but not to affect-based messages [33]. These results imply that the informative nature of a system’s self-tracked, sensor-based information that supports self-monitoring activities should appeal especially to individuals with high NFC. NFC has also been connected to intrinsic motivation, and it predicts intrinsic enjoyment and both self-perceived and behavioral motivation to engage in cognitive, effortful elaboration [64]. We hypothesize that NFC has a similarly positive influence on self-monitoring activity that provides data, information, and the opportunity to engage in conscious processing in terms of information related to the self:

H8: NFC moderates the relationship between self-monitoring and perceived persuasiveness.

##### Perceived Credibility and Perceived Persuasiveness

The role of source credibility, often in the form of source expertise, in persuasion is crucial in ELM and NFC research. It is considered a peripheral cue, and therefore individuals with low NFC should be more susceptible to processing such information. However, according to the multiple roles postulate, a variable such as credibility “...can have the same impact on judgements by different processes in different situations” [21]. Therefore, the credibility indicators of the system can also be processed in both ways. For example, the social influence strategy of authority [65], which is often referred to as a peripheral credibility cue, might also be processed through the central route among individuals with high NFC [66]. In general, credible sources are more persuasive; however, studies have indicated that peripheral cues are considered less meaningful when the argument is strong and personally relevant. In our research setting, we consider arguments to be mainly strong and personally relevant, which might partly suppress the effect of credibility. To evaluate the relevance of the aforementioned assumptions, we set the following hypothesis to address the role of NFC:

H9: NFC moderates the relationship between perceived credibility and perceived persuasiveness.

## Methods

### Measurement Instrument

We adapted scales that have been validated in several previous studies. Feedback, self-monitoring, perceived credibility, and perceived persuasiveness are all based on existing constructs developed to evaluate a PSD model [16]. The first two of these constructs were modified slightly to provide more details regarding self-monitoring system features. Therefore, they have also been named according to the PSD model principle instead of according to the category from which they are derived (dialog support and primary task support). Self-reported activity is a single-item measure developed for this study to query the subjective evaluation of perceived change in physical activity. To measure NFC, we used a shortened version of the NFC scale assessed in the study by Chiesi et al [67]. A detailed list of the measurement items and their exact wordings as used in this study are presented in Multimedia Appendix 1 [29,39-41,67].

### Data Collection

The questionnaire was implemented with the Webropol survey and reporting tool and sent to both students and employees of the University of Oulu, Finland, using mailing lists. The introductory text for the survey indicated that the respondents should be using a digital system that enables them to monitor their physical activity but that it does not have to be for that purpose only. We targeted users who had used the system for at least two months, which was also mentioned in the survey invitation. There were no other requirements or restrictions for participation, and the survey was fully anonymous.

The questionnaire consisted of 3 parts. The first part queried which systems the respondent used and how long they had used them to monitor their activity. The question regarding the estimation of current physical activity level compared with activity level before using the system was presented before the second part, which presented the theoretical measurement items. The final part collected respondent demographics. A detailed list of measurement items is presented in Multimedia Appendix 1. In terms of measurement items, 2 constructs were included on each page of the survey, and the pages were in the same fixed order for all respondents. The items for the 2 constructs on each page were randomly ordered. All the constructs were measured on a 7-point Likert scale, and the single-item measure for the subjective evaluation of the increase in physical activity was a scale of 5 statements that used typical 5-point Likert wordings.

During the 1-week survey period in October 2019, 261 responses were received. All the questions were mandatory; therefore, no data were missing. We removed cases that reported the use of systems that did not match our criteria (self-monitoring of physical activity). In addition, outlier responses were removed if they had a high likelihood of being faulty, such as when all the questions on a page (consisting of 2 constructs) had the same extreme value and the other values did not reflect similar evaluations of the system. It should be noted that we did not remove outliers based only on the values themselves, but also on highly inconsistent responses in general. The final sample consisted of 238 valid responses.

The choice of analysis method was structural equation modeling (SEM), namely partial least squares SEM (PLS-SEM). PLS-SEM is especially suitable for explorative research with non–normally distributed data [68,69]. It is also able to analyze complex models with a relatively small sample size [69]. However, for our model, the minimum sample size was achieved for the full sample and all subgroups that we derived from the sample. According to the study by Hair et al [69], the minimum sample should be 10 times the largest number of structural paths directed at a particular latent construct in the structural model, which in our model was 30. The collected data were analyzed using SPSS software (version 26.0; IBM Corp) and SmartPLS software (version 3.0; SmartPLS GmbH) [70].

## Results

### Demographic Information and Use of Systems

The final sample consisted of 238 valid responses. The basic demographics of the participants are presented in Table 1. The respondents were primarily women. Of the 238 respondents, 144 (60.5%) were aged below 30 years, 180 (75.6%) had an undergraduate or higher degree, and 99 (41.6%) had used a monitoring system for more than 2 years.

Altogether, the respondents used more than 20 different services, and 29.8% (71/238) provided the name of the specific tracker they used in addition to a mobile app (adding a tracker was not mandatory because many services can be used without an additional tracker). A few respondents used several devices such as the Ōura Ring (Ōura Health Oy), Vivofit (Garmin Ltd), or Polar M400 (Polar Electro) for activity tracking and a specific sports watch for measuring training sessions. The most used system was the Polar Flow app (92 users), which is used to support the use of Polar activity trackers and heart rate monitors. Many of these apps can be used with several different trackers, as is the case for apps such as Polar Flow (Polar Electro), Suunto (Amer Sports Oyj), Garmin (Garmin Ltd), and Fitbit (Fitbit LLC). Multimedia Appendix 2 illustrates the basic self-monitoring and feedback features of the 8 most common systems.

**Table 1:** Sample characteristics (N=238).

Variable and category			Participant, n (%)
**Gender**			
	Women	137 (57.6)	
	Men	100 (42)	
	Other	1 (0.4)	
**Age groups (years)**			
	19-29	144 (60.5)	
	30-39	48 (20.2)	
	40-49	27 (11.3)	
	≥50	19 (8)	
**Service use time (months)**			
	2-6	41 (17.2)	
	6-12	40 (16.8)	
	12-24	58 (24.4)	
	24-36	41 (17.2)	
	>36	58 (24.4)	
**Service (app name)**			
	Polar Flow	92 (38.7)	
	Apple Health	19 (8)	
	Sports Tracker	19 (8)	
	Suunto app	17 (7.1)	
	Ōura	16 (6.7)	
	Garmin Connect	14 (5.9)	
	Fitbit	13 (5.5)	
	Samsung Health	10 (4.2)	
**Education**			
	High school diploma	47 (19.7)	
	Vocational degree	11 (4.6)	
	Bachelor’s degree	82 (34.5)	
	Master’s degree	68 (28.6)	
	Doctor’s degree	30 (12.9)	

### Analysis of Distributions and Group Characteristics

In this section, we present the results of the statistical analysis of the collected data. First, we analyzed whether our results differed in relation to the NFC scores or other groups possibly relevant for this study. To study the distribution among the levels of NFC scores, we divided our sample into 3 proportions—low, moderate, and high NFC—using visual binning. The upper limit of the cut point was included in the group, and this resulted in groups with 84, 78, and 76 individuals, respectively. This three-group approach is partly aligned with the recommendation for system design and message tailoring provided in the study by Nikoloudakis et al [71]. This recommendation, however, prefers categorization using SD because it correctly identifies the nature of the measurement (most individuals fall in the moderate-level category), but our sample was too small for such a grouping.

**Figure 2:**
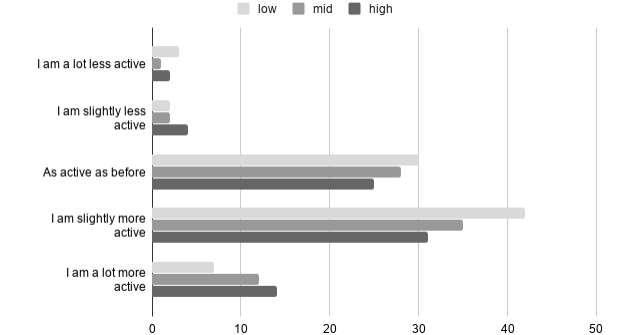
Self-reported activity after adopting the system in the need for cognition groups.

We also tested whether our theoretical measures were distributed equally across other groups such as the self-monitoring systems that the respondents used, duration of system use, age group, education, or gender. There were significantly different distributions among the NFC subgroups, genders, and systems used. The Kruskal-Wallis test showed that the NFC subgrouping significantly affected how individuals responded in terms of self-monitoring (Kruskal–Wallis *H*_2_=7.576; *P*=.02), feedback (Kruskal–Wallis *H*_2_=8.639; *P*=.01), perceived credibility (Kruskal–Wallis *H*_2_=17.463; *P*<.001), and perceived persuasiveness (Kruskal–Wallis *H*_2_=16.786; *P*<.001). In all the aforementioned tests, individuals with high NFC reported significantly higher mean scores than those in the low or moderate subgroups. For self-monitoring and feedback, pairwise comparison revealed a significant difference between the low- and high-NFC groups (*P*=.048 and *P*=.01, respectively). Perceived credibility and perceived persuasiveness differed in pairwise comparisons between both low and high (*P*<.001 and *P*=.001, respectively) and moderate and high (*P*=.02 and *P*=.001, respectively). The distributions of NFC were not significantly different for the self-reported activity levels. The frequencies of responses in the NFC subgroups are presented in Figure 2. Overall, 58.8% (140/238) of the respondents perceived that their activity was higher than before they started using the system.

Women evaluated their systems significantly higher in terms of perceived persuasiveness (Kruskal–Wallis *H*_2_=7.485; *P*=.02). The duration of system use, age group, and education all exhibited the same distributions across the subgroups. Table 2 presents the basic statistics of the full sample and the groups that showed differences, namely, the gender and NFC subgroups.

**Table 2:** Means (SDs) for variables per full sample and subgroups.

Variables	Value, mean (SD)					
	Full sample (N=238)	Women (n=137)	Men (n=100)	NFC^a^ group		
				Low (n=76)	Moderate (n=78)	High (n=84)
Self-monitoring	4.96 (1.20)	5.01 (1.24)	4.90 (1.15)	4.83 (1.17)	4.78 (1.26)	5.29 (1.11)
Feedback	4.83 (1.24)	4.83 (1.28)	4.82 (1.19)	4.65 (1.17)	4.69 (1.25)	5.17 (1.25)
Perceived credibility	5.33 (0.97)	5.35 (1.03)	5.29 (0.88)	5.06 (0.97)	5.29 (0.89)	5.66 (0.95)
Perceived persuasiveness	5.31 (1.16)	5.47 (1.13)	5.09 (1.18)	5.10 (1.17)	5.12 (1.17)	5.72 (1.04)
NFC	4.94 (0.91)	4.93 (0.96)	4.95 (0.84)	4.00 (0.57)	4.97 (0.15)	5.95 (0.45)
Self-reported activity	3.65 (0.85)	3.80 (0.76)	3.45 (0.94)	3.57 (0.83)	3.71 (0.81)	3.67 (0.93)

^a^NFC: need for cognition.

**Figure 3:**
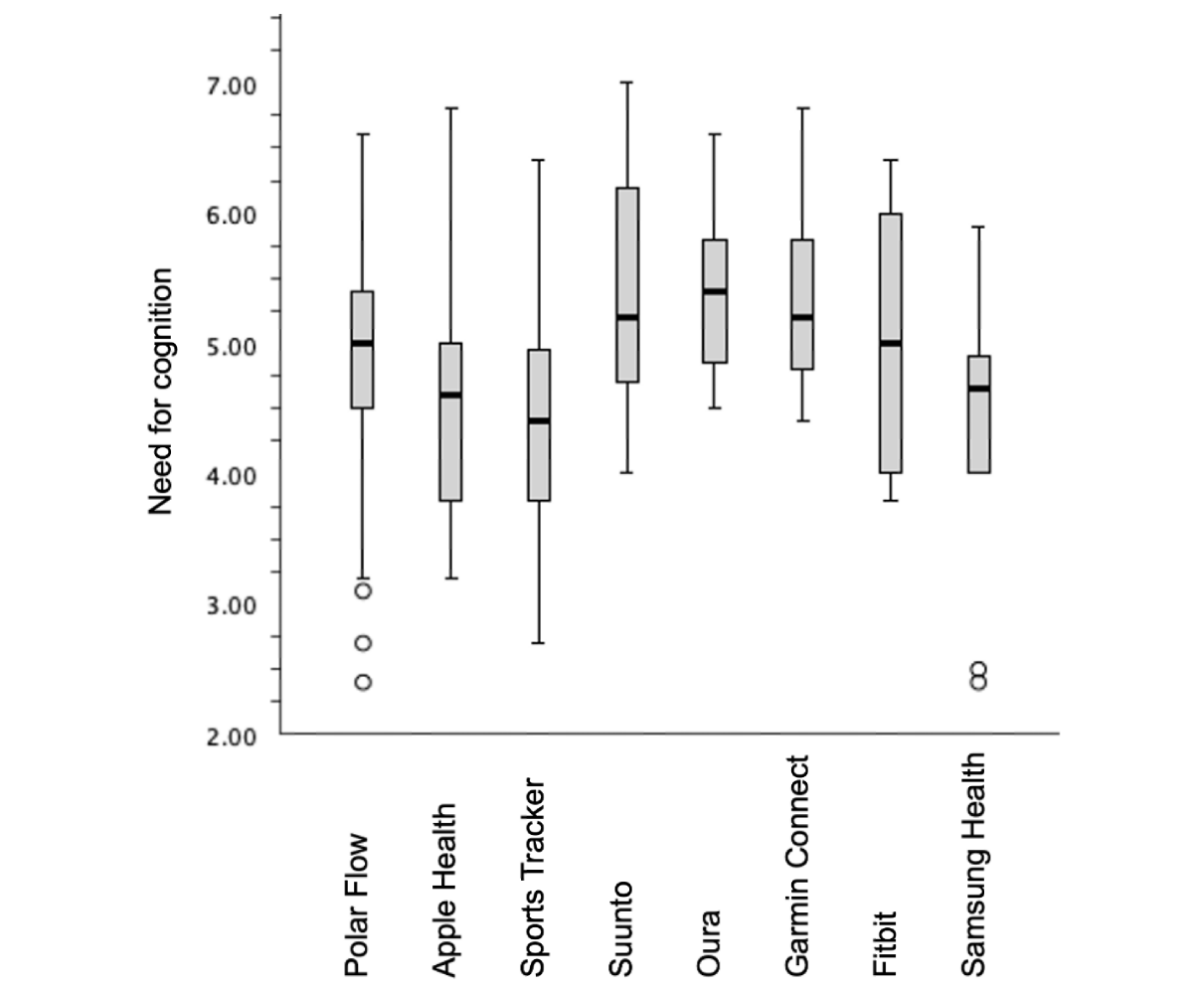
Distributions of need for cognition scores among users of top eight self-monitoring systems.

The distributions of measured theoretical constructs (self-monitoring, feedback, perceived credibility, and perceived persuasiveness) were the same across all 8 self-monitoring systems used. However, the distribution of NFC scores differed across the 8 systems (*H*_7_=21.709; *P*=.003). NFC differed significantly in one pairwise comparison, with the difference between Sports Tracker (Sports Tracking Technologies) and Ōura Ring users (*P*=.02) in terms of adjusted, Bonferroni-corrected values. Figure 3 shows the means and distributions of the NFC scores among the 8 most used systems. Sports Tracker users scored the lowest (mean and overall). Their NFC levels were similar to those of the users of the platform services Apple Health and Samsung Health. Dedicated health trackers (Polar, Suunto, Ōura, Garmin Connect, and Fitbit) scored marginally higher overall.

### Structural Equation Modeling

#### Measurement Model

PLS-SEM analysis comprises 2 steps. First, the measurement model is assessed by analyzing the relationship of each indicator with its corresponding construct. All our constructs were reflective. Internal consistency reliability was measured using Cronbach α and composite reliability, as presented in Table 3. Composite reliability varies between 0 and 1, with higher values indicating higher levels of reliability. It is generally interpreted in the same way as Cronbach α, and values between 0.70 and 0.90 can be regarded as satisfactory [72]. In our data, composite reliability was acceptable for all the constructs after we followed the instructions provided in the study by Hair et al [73] and removed some items. The full set of our original and final measurement items is presented in Multimedia Appendix 1, with references to relevant studies and item loadings.

**Table 3:** Properties of latent variables, including Cronbach α, composite reliability, and average variance extracted.

Variables	Cronbach α	CR^a^	AVE^b^	NFC^c^	CRED^d^	PEPE^e^	FEEDB^f^	SELFM^g^
NFC	.845	0.889	0.616	*0.785* ^h^	—^i^	—	—	—
CRED	.862	0.901	0.645	0.241	*0.803*	—	—	—
PEPE	.862	0.906	0.706	0.237	0.645	*0.840*	—	—
FEEDB	.805	0.885	0.720	0.185	0.521	0.563	*0.848*	—
SELFM	.773	0.868	0.687	0.168	0.589	0.657	0.670	*0.829*

^a^CR: composite reliability.

^b^AVE: average variance extracted.

^c^NFC: need for cognition.

^d^CRED: perceived credibility.

^e^PEPE: perceived persuasiveness.

^f^FEEDB: feedback.

^g^SELFM: self-monitoring.

^h^The variables in italics show the square root of average variance extracted and interconstruct correlations.

^i^Not applicable.

Discriminant validity was assessed using the Fornell-Larcker criterion: the values in italics in Table 3 showing the square root of average variance extracted should be higher than the interconstruct correlations. We omitted one item each from the self-monitoring and feedback constructs to tackle a heterotrait-monotrait ratio that was initially above 0.9. These omissions resulted in a satisfactory heterotrait-monotrait ratio level. The loading of each item of the constructs and the cross-loadings that validate the discriminant validity of our constructs are presented in Multimedia Appendix 3.

#### Structural Model and Hypothesis Testing

The second part of PLS-SEM, evaluation of the structural model, represents the underlying theory of the path model and allows for the determination of how well the empirical data collected supports the theory-derived hypotheses. The key results are obtained by defining the path coefficients and explained variances (*R*^2^ values). We followed the recommendation provided in the study by Hair et al [73] and opted not to use the goodness-of-fit criterion for PLS-SEM. We used the complete bootstrapping method with 5000 resamples and parallel processing with no sign changes. The CI method was the two-tailed bias-corrected and accelerated bootstrap (default setting). For the moderation hypotheses, we generated interaction terms with NFC as a continuous moderator and used the product indicator calculation method and mean-centered product term generation as recommended in the study by Hair et al [73].

The results of the hypothesis testing for the research model are presented in Table 4. All the basic hypotheses were supported by the full sample. Effect size (Cohen *f*^2^) was considered large if it was above 0.35. Nonsignificant (below 0.02) effect sizes have been removed from the table. There was no significant relationship between feedback features and perceived persuasiveness of systems for men, which was the only hypothesis not supported for both genders. Analysis of the 3 NFC groups revealed that feedback had a positive influence on perceived persuasiveness only for individuals with high NFC. Perceived credibility had a positive effect on perceived persuasiveness in all the groups except the one with individuals with high NFC, with a large effect size for the moderate-NFC group. Finally, perceived persuasiveness was positively related to self-reported activity evaluations for all groups except the one with individuals with low NFC, with a large effect size only for individuals with high NFC. To summarize, of the 6 basic hypotheses analyzed, 3 indicated significant differences among the NFC groups. The relationship between feedback and self-monitoring was the most stable path, with all group hypotheses supported with *P*<.001. The respondents also reported their perceived physical activity level compared with their activity level before using the system. There was a significant relationship between perceived persuasiveness and self-reported activity for all groups, except for individuals with low NFC. However, the relationship had a large effect size only for individuals with high NFC.

**Table 4:** Results of hypothesis testing for the full sample and representative groups (N=238).

Hypothesis and group		Path	*t* test (*df*)	*P* value	Cohen *f*^2^	Support
**H1: Feedback functionality is positively related to the perceived persuasiveness of the system**						
	Full sample	0.140	2.188 (8)	.04	0.023	Yes
	Women	0.158	1.931 (8)	.05	0.028	Yes
	Men	0.161	1.677 (8)	.13	0.032	No
	Low NFC^a^	0.018	0.162 (8)	.90	—^b^	No
	Moderate NFC	0.159	1.628 (8)	.36	0.045	No
	High NFC	0.218	2.047 (8)	.03	0.049	Yes
**H2: Feedback functionality is positively related to self-monitoring features**						
	Full sample	0.499	8.200 (8)	<.001	0.385	Yes
	Women	0.503	5.811 (8)	<.001	0.421	Yes
	Men	0.502	7.057 (8)	<.001	0.356	Yes
	Low NFC	0.662	8.735 (8)	<.001	0.844	Yes
	Moderate NFC	0.447	3.669 (8)	<.001	0.277	Yes
	High NFC	0.414	4.494 (8)	<.001	0.283	Yes
**H3: Self-monitoring is positively related to the user’s perceived persuasiveness evaluation**						
	Full sample	0.348	4.494 (8)	<.001	0.125	Yes
	Women	0.234	2.361 (8)	.02	0.054	Yes
	Men	0.478	4.325 (8)	<.001	0.271	Yes
	Low NFC	0.406	3.065 (8)	.003	0.146	Yes
	Moderate NFC	0.354	3.486 (8)	<.001	0.205	Yes
	High NFC	0.400	2.631 (8)	.01	0.127	Yes
**H4: The perceived credibility of the system has a positive influence on self-monitoring features**						
	Full sample	0.329	5.013 (8)	<.001	0.167	Yes
	Women	0.348	3.862 (8)	<.001	0.202	Yes
	Men	0.306	3.564 (8)	<.001	0.133	Yes
	Low NFC	0.212	2.368 (8)	.02	0.087	Yes
	Moderate NFC	0.306	2.363 (8)	.02	0.123	Yes
	High NFC	0.451	4.619 (8)	<.001	0.336	Yes
**H5: Perceived credibility has a positive relationship with perceived persuasiveness**						
	Full sample	0.352	4.870 (8)	<.001	0.168	Yes
	Women	0.446	5.620 (8)	<.001	0.279	Yes
	Men	0.220	1.848 (8)	.07	0.070	Yes
	Low NFC	0.443	3.596 (8)	<.001	0.312	Yes
	Moderate NFC	0.476	6.023 (8)	<.001	0.475	Yes
	High NFC	0.132	0.921 (8)	.36	0.019	No
**H6: Perceived persuasiveness has a positive influence on self-reported level of activity after adopting a self-monitoring system**						
	Full sample	0.335	5.484 (8)	<.001	0.127	Yes
	Women	0.260	3.331 (8)	.001	0.072	Yes
	Men	0.372	4.026 (8)	<.001	0.162	Yes
	Low NFC	0.182	1.420 (8)	.16	0.034	No
	Moderate NFC	0.311	2.745 (8)	.006	0.107	Yes
	High NFC	0.511	6.429 (8)	<.001	0.355	Yes
**H7: NFC moderates the relationship between feedback and perceived persuasiveness. The positive influence of feedback is stronger for individuals with high NFC because of the informative nature of feedback in self-monitoring systems**						
	Full sample	–0.048	0.851 (8)	.40	0.025	No
	Women	–0.066	1.421 (8)	.16	0.052	No
	Men	0.027	0.360 (8)	.72	0.007	No
**H8: NFC moderates the relationship between self-monitoring and perceived persuasiveness**						
	Full sample	–0.040	0.822 (8)	.41	0.016	No
	Women	–0.083	2.875 (8)	.004	0.074	Yes
	Men	0.047	0.634 (8)	.53	0.015	No
**H9: NFC moderates the relationship between perceived credibility and perceived persuasiveness**						
	Full sample	–0.090	1.363 (8)	.17	0.051	No
	Women	–0.098	2.077 (8)	.04	0.080	Yes
	Men	0.126	0.871 (8)	.38	0.124	No

^a^NFC: need for cognition.

^b^Nonsignificant values are omitted.

**Figure 4:**
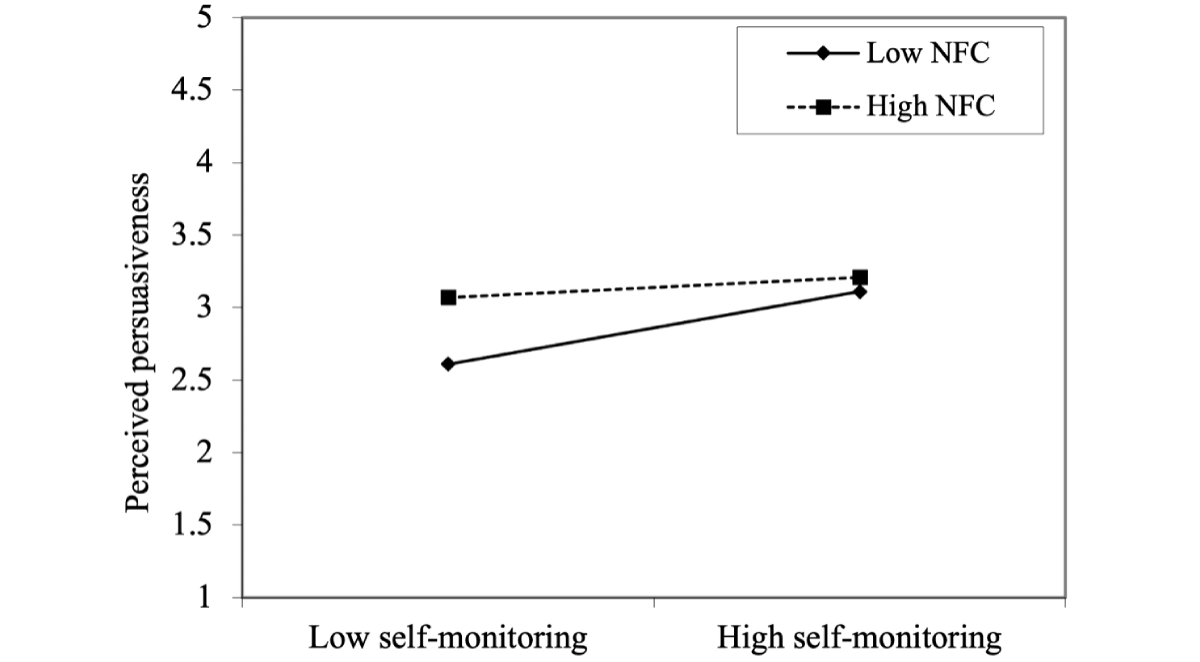
Simple slope showing the need for cognition moderation effect for self-monitoring’s influence for perceived persuasiveness among women (hypothesis H8). NFC: need for cognition.

**Figure 5:**
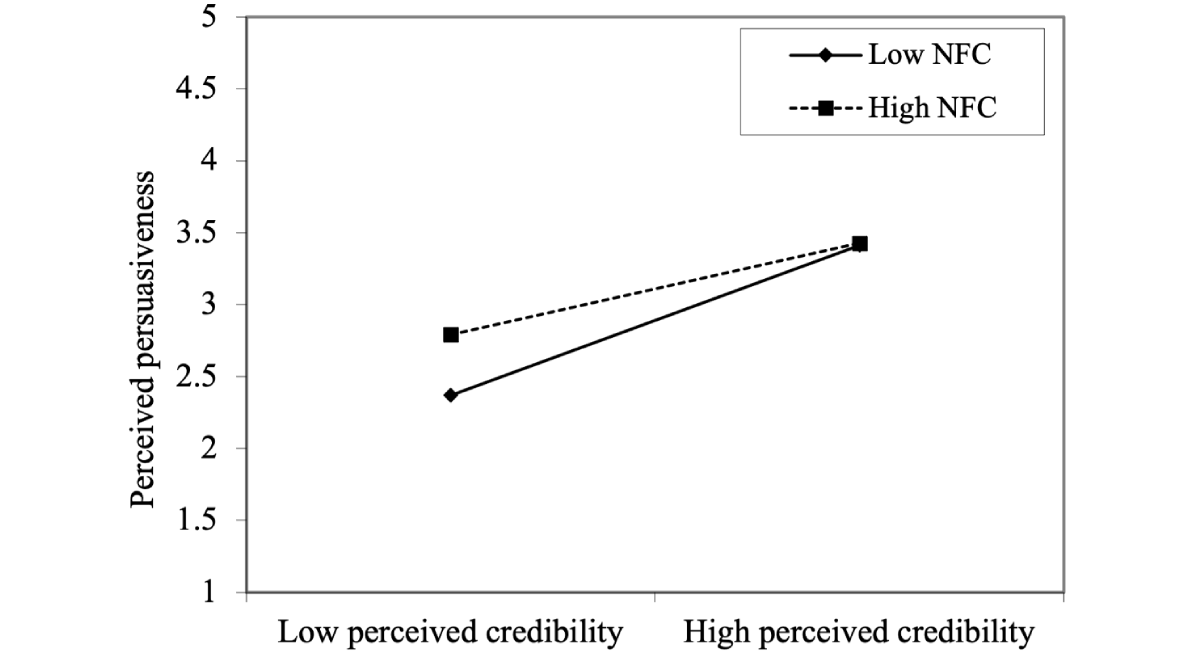
Simple slope showing the need for cognition moderation effect for perceived credibility’s influence for perceived persuasiveness among women (hypothesis H9). NFC: need for cognition.

Two of the three moderation hypotheses were supported, but only for women. Moderator path coefficients were negative for women and positive for men. Simple slopes were drawn for the supported hypotheses for women. In terms of perceived persuasiveness of the system, self-monitoring features made no difference to those with high NFC (Figure 4); however, for women with low NFC, high self-monitoring increased the perceived persuasiveness of the system. For both women with low NFC and women with high NFC, high perceived credibility of the system increased the perceived persuasiveness of the system (Figure 5). However, this effect was stronger for individuals with low NFC, although it reached levels of persuasiveness similar to those among women with high NFC when the credibility was high.

Analysis of *R*^2^ values (Table 5) indicated that, altogether, feedback, self-monitoring, and perceived credibility explained 54.4% of the variance in the perceived persuasiveness of the system. This value peaked among individuals with moderate NFC, where self-monitoring and perceived credibility explained approximately 64% of the perceived persuasiveness construct. For individuals with high NFC, feedback and self-monitoring explained 47.3% of perceived persuasiveness. Variance in the self-monitoring construct was best explained for individuals with low NFC (63.8%) and least for the moderate-NFC group (41.1%). Finally, the single-item construct, self-reported activity, was significant only for men with high NFC and the high NFC group. Overall, both exogenous constructs (perceived persuasiveness and self-monitoring) revealed more differences within the NFC groups than between the genders.

**Table 5:** Variances explained as percentages (*R*^2^; N=238).

Construct	Variance (%)					
	Full sample, variance	Women, variance	Men, variance	NFC^a^ group, variance		
				Low	Moderate	High
Perceived persuasiveness	54.4	54.5	59.4	59.2	63.9	47.3
Self-monitoring	52.8	54.7	51.8	63.8	41.1	58.2
Self-reported activity	11.3	6.8^b^	14	3.8^b^	9.7^b^	26.2

^a^NFC: need for cognition.

^b^Nonsignificant value.

## Discussion

### Principal Findings

This study explored the relationship between the individual trait of NFC and commercial self-monitoring systems. In addition, it aims to understand whether this trait shows promise in designing systems for self-monitoring. We found that the respondents evaluated all studied features significantly differently based on their level of NFC, and, to some extent, there were differences in their system choices. Perceived credibility did not contribute to persuasiveness for individuals with high NFC; for these individuals, high persuasiveness was constructed based on self-monitoring and feedback. For individuals with low and moderate NFC, perceived credibility also influenced the persuasiveness of the system. There was no relationship between perceived persuasiveness and self-reported activity level among individuals with low NFC, but the effect was large for individuals with high NFC. In addition, it seems that the individuals’ system choices reflected their intrinsic motivations to engage with rich sources of data, exemplified by an overall lower level of NFC among users of smartwatch systems and a higher level of NFC among dedicated fitness device users.

SEM revealed several differences among the NFC groups. Our study reports that feedback features increased the perceived persuasiveness of the system only for individuals with high NFC, and the effect was small. Previous research indicated that NFC is a trait that might motivate individuals to seek feedback. The recent study by Vaughan-Johnston and Jacobson [62] reported 2 studies that focused on preferences for different types of self-relevant feedback. NFC was related to an assessment type of feedback, and this relationship was stronger for individuals with high NFC. In our study, the relationship between feedback and self-monitoring features, in contrast, was strong among all NFC subgroups, and the effect size for individuals with low NFC (0.844) was exceptionally large. These results are in line with previous research suggesting that individuals with low NFC tend to take feedback more literally [74], without extensive elaboration; therefore, feedback and self-monitoring features form the strongest connection among those with low NFC. On the basis of this insight, it seems that individuals with high NFC do not accept feedback as is but use it as a data source to elaborate on the issue.

There was a prominent difference among the NFC groups in terms of the role of perceived credibility in forming the persuasiveness of the system. This relationship was strong in both the low- and moderate-NFC groups but nonexistent in the high-NFC group. This is in line with previous research that states that individuals with low NFC are more susceptible to persuasion using peripheral cue variables such as the 2 dimensions of credibility: expertise and trustworthiness [53,75,76]. Overall, the influence of perceived credibility on the users’ evaluation of a primary task type of feature, in this case self-monitoring, was stronger for individuals with high NFC, implying that the overall perception of source credibility for individuals with high NFC might be mediated through their evaluation of self-monitoring tasks rather than through more general credibility cues such as trustworthiness or source credibility.

Perceived persuasiveness is usually explained by the direct effect of self-monitoring, which lends support for its innate persuasive nature and ability to evoke goal-directed actions. This was strongest for individuals with moderate NFC and weakest for individuals with high NFC, for whom the direct effect of perceived credibility was lacking. Individuals with high NFC seemed to derive persuasiveness also from direct interaction with the feedback features instead of more peripheral source credibility variables. In general, both credibility and persuasiveness evaluations were high in the full sample, indicating positive attitudes toward these systems in general. In addition, these scores were significantly higher among individuals with high NFC, which is to be expected based on the nature of the NFC trait. Considering the length of time our respondents had used these systems, prolonged use itself suggests user satisfaction. The repetitive nature of the interaction with the system might also contribute to high persuasiveness because (moderate) repetition has been identified as elaboration-enhancing and persuasive [77-80]. Repeating weak arguments would most likely not support long-term engagement with the system; therefore, our findings might suggest that, overall, users of these systems perceive the messages that the systems deliver as strong arguments, which increases the perceived persuasiveness in prolonged use. However, perceived behavior change, measured by self-reported activity after adopting the system, is not related to perceived persuasiveness among individuals with low NFC.

Although NFC is considered gender-neutral [30], our moderation hypotheses resulted in significant moderation only for women. To our knowledge, gendered moderation has not been previously reported. There were no significant differences in the systems the women used or in NFC values, but the women evaluated their systems as significantly more persuasive than the men. For women with low NFC, the evaluation of self-monitoring clearly increased the persuasiveness of the system such that a high rate of use of the self-monitoring feature resulted in a more persuasive system. There was no similar effect for women with high NFC, who rated persuasiveness very similarly, regardless of how they perceived self-monitoring. The same finding was present also for hypothesis H3. Altogether, self-monitoring and credibility features increased the perceived persuasiveness for women with low NFC to the same degree as for their high-NFC counterparts, which could imply that these features can improve attitudinal responses toward these systems.

### Comparison With Prior Work

There is very little previous research to compare our findings with, in terms of NFC and use of self-monitoring technologies. The only study available [81] used a short German version of an NFC scale with 4 items [82]. The German version is based on the original 34-item scale [29], and we used a 10-item scale [67] that discarded 2 of the items in the German scale. One item included in the German scale was removed from our SEM analysis because of low loading, leaving our study to share only one item with the study by Attig et al [81]. Our study is, to our knowledge, the first to report a significant relationship between NFC and commercial self-monitoring tools. When we compared our study with previous research on tailoring in the health domain, we found that a few studies had used three-item questionnaires [83,84]. All these 3 items were included in our questionnaire, which showed good internal consistency, but 2 of them were discarded from the SEM analysis because of insufficient loadings.

Self-monitoring systems that are designed specifically for self-tracking and that collect several different metrics and present a complex set of indicators to the user to reflect upon (in this study, examples of such systems are Polar, Suunto, and Ōura) seem to be favored by users with high NFC. Exploring new or different systems can itself be considered a cognitively engaging activity [85], and a tendency to seek support and solutions for personal health issues from sources such as measuring devices fits well with previous research on individuals with high NFC. Users of self-monitoring apps without a dedicated fitness or activity tracker, exemplified by Apple Health, Sports Tracker, and Samsung Health, scored generally lower in terms of NFC. This might be a result of self-tracking mainstreaming through smartwatches and mobile sensors. Many individuals end up tracking their activity without a specific aim to do so because the functionalities are available through multipurpose devices. The wide range of NFC scores for Fitbit, which has tracking devices for both low and high ends of technical and functional requirements, also supports the idea that NFC might function at the system selection level, and tailoring system features and content might not be the most obvious tailoring targets. In terms of volitionally used self-selected systems and the information they present to users, the subjective nature of complexity [32] poses interesting questions regarding preferred systems, especially because individuals with high NFC restrain themselves from engaging in effortful mental work when a message is perceived to be simple.

There is surprisingly little published research on NFC as an individual difference affecting system use. We identified differences in system evaluations in terms of NFC in every software feature construct that we measured. This implies that differences in subjective evaluations of features might indeed be a facet that allows access to differences in information processing among NFC groups. There are several possible explanations for why individuals with high NFC seem to evaluate their systems more favorably in terms of persuasive features. Previous research has indicated that individuals with high NFC are less supportive of punitive measures [86], and the study suggests that this might be due to their tendency to form complex attributions for human behavior. This would be based on their innate motivation to engage in effortful thinking about the causes of human behavior in general. This could apply to the complexity of human behavior around physical activity. Perhaps individuals with high NFC do not expect simple and actionable insights to guide their behaviors, as individuals with low NFC do, but instead value data and raw information that they can use in their own thinking process. The extensive amount of information provided by these systems may also allow for greater task complexity, which individuals with high NFC prefer [32]. If self-monitoring systems feed their enjoyment in working out their own behavior with data, it might be intrinsically motivating and persuasive for them.

Alternatively, or in addition, individuals with high NFC might also have a significant amount of affinity for technology interaction [85]. The data that the systems provide smoothen their system evaluations, and individuals with high NFC accept more discrepancies or shortcomings from their systems. Individuals with high NFC might also form stronger engagement with the device concerned because of their interest in data: the more a monitoring system is used, the more data are collected for further elaboration. Positive evaluations of the system might also be consistent with a high amount of system use, but our data or findings do not address this issue because we did not measure system use frequency. To summarize our findings, higher levels of NFC were associated with higher system evaluations; for perceived credibility and persuasiveness, this trend is so strong that it separates the high-NFC group from both lower-NFC groups. This finding cannot be explained merely by differences among the systems because there was only one pair of systems (Sports Tracker vs Ōura) that differed from each other in terms of the NFC levels of the users.

### Practical Implications

NFC has been studied widely for several decades, and its basic characteristics are well established. However, in the context of systems design, it is usually considered a trait for tailoring or personalization. Our results, based on systems that individuals with different levels of NFC had selected themselves, imply that targeting NFC might not be feasible for all types of systems. This is because the selection procedure might have excluded some systems from certain levels of NFC. For example, simple ones with basic functions might not be perceived as worth a further look by individuals with high NFC. Therefore, there is no need to tailor these systems for individuals with high NFC. This selection process might be influenced by brand image and communication, peers, peer reviews, and social media, which are not always controllable by those in charge of product development. However, practitioners might want to consider whether their marketing and product information communication fits with a desire to engage with problem solving and an interest in rich data. Similarly, products that seem to persuade users with high NFC might be equally useful and interesting to individuals with lower levels of NFC when accompanied by strong credibility cues and clearly designed self-monitoring features. For example, the very strong relationship between feedback and self-monitoring among individuals with low NFC implies that they might appreciate actionable and unambiguous support from the system and wish not to rely on their own cognitive work. The extensive communication of advanced product characteristics might not resonate well with their goals, and they may then choose other systems.

Our results also indicate that regardless of the NFC level, perceived credibility is important for the persuasiveness of these systems. It is, however, constituted slightly differently based on the level of NFC. For individuals with high NFC, its impact is transferred through self-monitoring activity and feedback features and might be more akin to a continuous evaluative process to validate the arguments that the system presents case by case. For individuals with low NFC, strong credibility might be more akin to an indication that the system is overall a good choice and should be used because its impact mainly targets overall persuasiveness instead of self-monitoring activity itself. Although our work does not allow elaboration likelihood or strength considerations, the results may be an indication of the use of source credibility as a cue for peripheral processing or as a variable whose validity is evaluated in daily activities through more effortful processing.

### Limitations and Directions for Future Research

Our results should be interpreted against the background of some limitations. Our sample was collected in a university setting using only English. Although university students in Finland, including native Finnish students, are generally fluent in English, this might have biased our sample toward those with higher-than-average NFC because (high) NFC is positively correlated with verbal information processing [30]. We aimed to decrease this bias by including the upper limit value in the NFC groups that we formed with visual binning. This resulted in relatively larger groups of individuals with moderate and high NFC.

The self-selection of systems also has implications for the generalizability of our findings. Most likely, our sample comprised individuals who are interested in health technology and, overall, have higher-than-average satisfaction with their systems. Our findings are therefore relevant for natural use settings but less so for studies where users cannot choose their systems freely or use them fully voluntarily. Consequently, we consider that our work advances the field of self-help tools used outside of the health care sector and without moderation by an outside party. The levels of NFC in the general population in Finland and in other commercial system users would help to address the amount of self-selection bias in our study and guide future data collection. It is also noteworthy that we did not measure self-selection but assumed that individuals more often select and buy these devices themselves. In addition, our participants were considered healthy individuals because health status information was not collected. However, a limitation of our data is that these issues were not addressed in the questionnaire or recruitment. Future studies might focus on individuals with different chronic conditions to understand if NFC has a relationship with some health issues.

In self-monitoring systems, feedback features are often embedded in self-monitoring activity itself, for example, as a chart that is updated according to progress during the day. Therefore, it is possible that the self-monitoring construct we used also embeds some of the influence of feedback, although the constructs are independent. Although we phrased the questions in a way that would reflect more prominent feedback features, it is possible that the otherwise theoretically sound approach to defining feedback and self-monitoring as separate BCTs is not fully applicable to self-monitoring systems. Future research should develop new scales to measure theory-based characteristics of feedback and self-monitoring in relation to both behaviors and outcomes of behaviors to shed additional light on the relationship among these BCTs [2] in actual, implemented systems.

In addition, the use of several different types of physical activity self-monitoring systems might have caused additional heterogeneity in our results. We did not find statistically significant differences among the top 8 systems in terms of theory-driven system features, but this might be due to the low numbers of users in several systems. Although these types of commercial systems are currently rather similar in their feature sets [38] and we measured only the basic features, some trends might have been partly caused by the details of different systems. Future studies should seek to replicate our findings with single self-selected system users or compare different software implementations in behavior change features. Such studies could also go deeper into implementation details and analyze where the actual differences among NFC levels, and especially among genders in terms of NFC, arise. For example, previous studies have suggested that the differences may lie in visual perception [87], or they may be due to the differences in elaboration style or interaction with, and perception of, the features themselves [88,89].

### Conclusions

Our study reported insights into a widely studied personality trait, the NFC, among users of commercial self-monitoring tools. In contrast to most of the research in the field of persuasion that focuses on this trait for attitude or behavior change, we used it to understand how individuals choose and use systems in a natural use environment. NFC does affect both the selection and use of systems, but the nature of the findings indicates that extensive aims to tailor content based on this trait might not always be a feasible approach. Instead, some features can also enable individuals with low NFC to benefit from these systems. However, the availability of different commercial systems might itself be a tool for tailoring, and individuals choose systems based on their innate characteristics.

Regardless of the apparent relevance of NFC to engaging with personally relevant data collection with self-monitoring tools, this paper is, to our knowledge, the first to report the role of NFC among self-monitoring users of wearables. Our data demonstrate that NFC as a trait that differentiates information processing has several implications for the selection, design, and tailoring of self-monitoring systems and their use in health interventions.

## Appendix

Multimedia Appendix 1 Survey instrument.

Multimedia Appendix 2 Short introductions of the eight most used self-monitoring systems of this study.

Multimedia Appendix 3 Cross-loadings for measurement items of the study.

## Abbreviations

BCT: behavior change technique
ELM: elaboration likelihood model
NFC: need for cognition
PLS-SEM: partial least squares structural equation modeling
PSD: persuasive systems design
SEM: structural equation modeling
