# Supplementary material for: Need for Cognition Among Users of Self-Monitoring Systems for Physical Activity: Survey Study
Source: JMIR Form Res. 2021 Oct 14;5(10):e23968. doi: 10.2196/23968 (PMC8554677; doi:10.2196/23968)
Supplement: Multimedia Appendix 1 [file formative_v5i10e23968_app1.pdf]

Multimedia Appendix 1.

Measurement items of the study survey instrument with their original source, wordings and loadings for structural equation modelling.

Construct	Measurement items	Loading
**Self-monitoring** Modified for this study based on and Lehto & Oinas-Kukkonen, 2015; Lehto, Oinas-Kukkonen, & Drozd, 2012; Lehto, Oinas-Kukkonen, Pätiälä, et al., 2012	The system helps me to set goals regarding my activity.	0.825
	The system helps me in keeping track of my progress.	0.843
	The system helps me to see how I progress towards my goals. DELETED to decrease heterotrait-monotrait ratio.	-
	The system helps me to understand where I am and where I want to be.	0.818
**Feedback** Modified for this study based on Lehto & Oinas-Kukkonen, 2015; Lehto, Oinas-Kukkonen, & Drozd, 2012; Lehto, Oinas-Kukkonen, Pätiälä, et al., 2012	The system gives me feedback that I can easily act on.	0.867
	The system gives me personally relevant feedback.	0.840
	The feedback provided by my system usually comes at the right time.	0.839
	The feedback that I receive encourages me. DELETED to decrease heterotrait-monotrait ratio.	-
**Perceived credibility** Developed based on	Overall, information that the system presents to me is accurate.	0.839
	I consider the system trustworthy.	0.832

and Lehto & Oinas-Kukkonen, 2015; Lehto, Oinas-Kukkonen, & Drozd, 2012; Lehto, Oinas-Kukkonen, Pätiälä, et al., 2012	Generally speaking, the measurements the system shows are believable.	0.820
	The information presented to me instils confidence.	0.805
	This system is created by professionals.	0.715
**Perceived persuasiveness** (Lehto, Oinas-Kukkonen, & Drozd, 2012)	In my opinion, the system is beneficial.	0.837
	Using the system has an effect on my physical activity.	0.858
	In my opinion, the system is convincing. DELETED due to low loading score.	-
	The system has an influence on me.	0.877
	The system makes me reconsider my physical activity habits.	0.787
**Need for Cognition** Original scale by Cacioppo and Petty (1982), the shortened version by Chiesi et al. (2018) used in this study.	*I would prefer complex to simple problems.* DELETED due to low loading score.	-
	I like to have the responsibility of handling a situation that requires a lot of thinking.	0.808
	Thinking is not my idea of fun. DELETED due to low loading score.	-
	I would rather do something that requires little thought than something that is sure to challenge my thinking abilities. DELETED due to low loading score.	-

	I try to anticipate and avoid situations where there is likely a chance I will have to think in-depth about something. DELETED due to low loading score.	-
	I find satisfaction in deliberating hard and for long hours.	0.749
	The idea of relying on thought to make my way to the top appeals to me.	0.818
	I really enjoy a task that involves coming up with new solutions to problems.	0.774
	I prefer my life to be filled with puzzles that I must solve.	0.775
	I would prefer a task that is intellectual, difficult, and important to one that is somewhat important but does not require much thought. DELETED due to low loading score.	-
Self-reported activity after adopting the system<br>Single-item measure developed for this study	Compared to your physical activity before you started using application, how would you rate your physical activity now when you are using the application? This question refers to your own estimation.	
	I am a lot less active	-
	I am slightly less active	
	As active as before	
	I am slightly more active	

	I am a lot more active	
