# Supplementary material for: Need for Cognition Among Users of Self-Monitoring Systems for Physical Activity: Survey Study
Source: JMIR Form Res. 2021 Oct 14;5(10):e23968. doi: 10.2196/23968 (PMC8554677; doi:10.2196/23968)
Supplement: Multimedia Appendix 2 [file formative_v5i10e23968_app2.pdf]

Multimedia Appendix 2.

Brand<br>Name of the mobile application	Self-monitoring features	Feedback features
Polar Electro<br>Flow application<br>(iOS, Android)<br><br>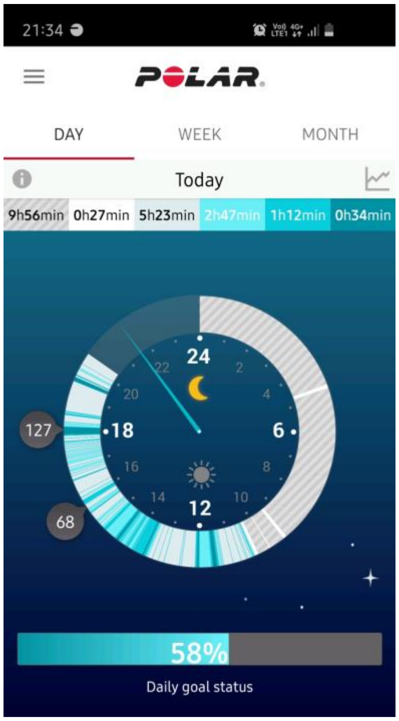	Automatic measuring of activity (e.g. steps, distance, active time, calories) and sleep (several metrics).<br><br>Presents statistics of all measured issues with different time-frames, e.g. weekly and monthly views.<br><br>Several advanced features depending on the sensors available in the wrist device.<br><br>Training features record rich data, including heart rate, pace, distance etc.<br><br>Works only with Polar wrist devices.	Activity presented as percentages of the goal, both in app (see figure) and in wrist device.<br><br>Activity levels are shown as time spent in each level and how they are located in the day (mobile app).<br><br>Inactivity alerts and alerts to notify that the goal of daily activity has been reached (both in app and in wrist device).<br><br>Feedback contains also verbal praise.

Brand<br>Name of the mobile application	Self-monitoring features	Feedback features
Apple Health<br>(iOS)<br>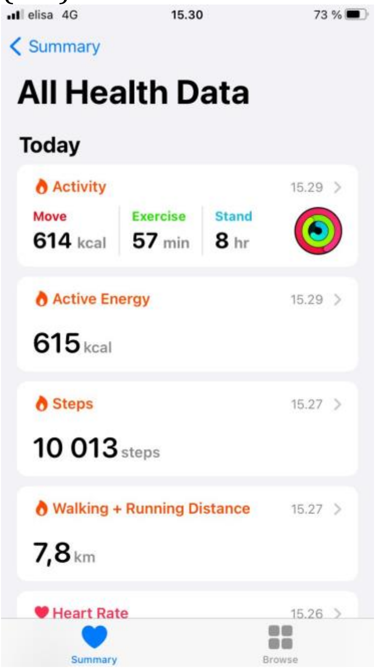	Automatic measuring of activity (steps, exercise, inactivity, calories etc.) and sleep (several metrics). Some features require iWatch as tracker.<br><br>Presents statistics of activities with different time-frames, e.g. weekly and monthly views.<br><br>Apple Health is an application that aggregates different types of data, some of them collected via 3rd party software or iWatch applications.<br><br>Basic sensing data is provided by phone sensors and some features of the app function without trackers.	Activity is presented as circles, both in app and in wrist device.<br><br>Both the wrist device and mobile application shows current status of each measured parameter.<br><br>The user can set goals when iWatch is used. Both app and iWatch give feedback on goal attainment.

Brand<br>Name of the mobile application	Self-monitoring features	Feedback features
Sports Tracker<br>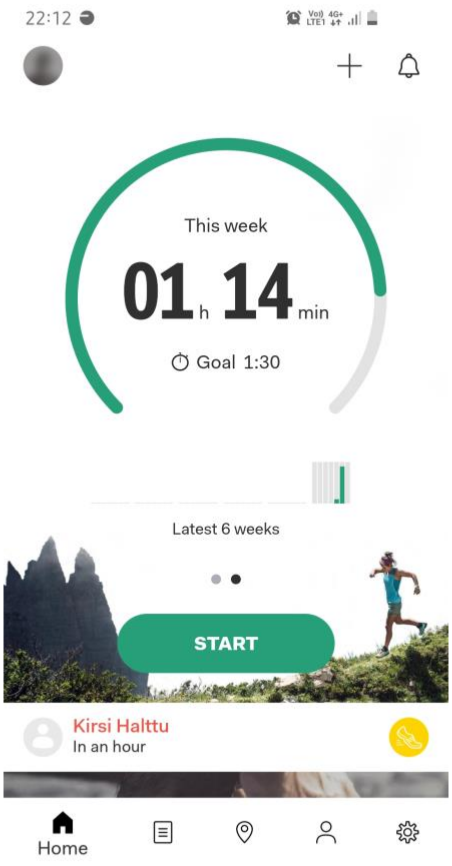	Measures activity when the training/recording session is started in the application.<br><br>Measures pace and distance, and uses map views.<br><br>Presents statistics of activities with different time-frames, e.g. weekly and monthly views.	Displays goal progress in amount of activity sessions tracked.<br><br>Enables setting goals and gives feedback on progress towards goals (see figure).

Brand<br>Name of the mobile application	Self-monitoring features	Feedback features
Suunto<br>Suunto application<br>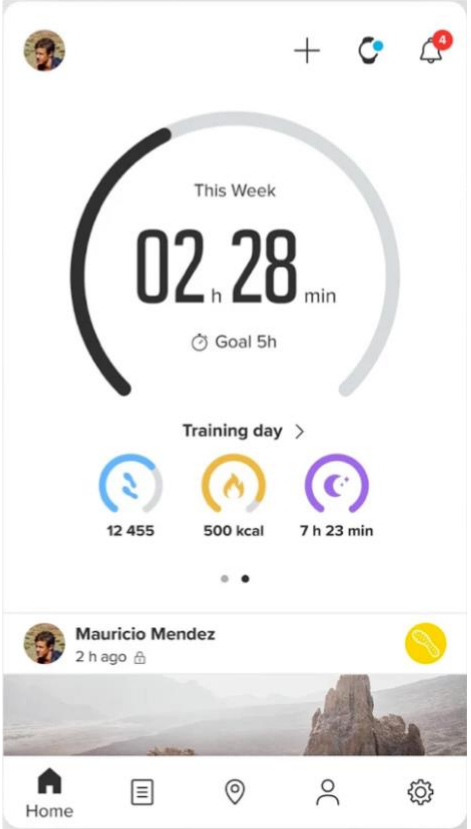	Automatic measuring of activity (e.g. steps, distance, active time, calories) and sleep (several metrics).<br><br>Presents statistics of all measured issues with different time-frames, e.g. weekly and monthly views.<br><br>Several advanced features depending on the sensors available in the wrist device.<br><br>Training features record rich data, including heart rate, pace, distance etc.<br><br>Works only with Suunto wrist devices.	Presents daily activity and sleep scores in the main page of the mobile app and in the wrist device.<br><br>Enables setting goals and monitoring goal progress (both mobile app and device).<br><br>Gives feedback on goal attainment.

Brand<br>Name of the mobile application	Self-monitoring features	Feedback features
Oura<br>Oura application (iOS, Android)<br>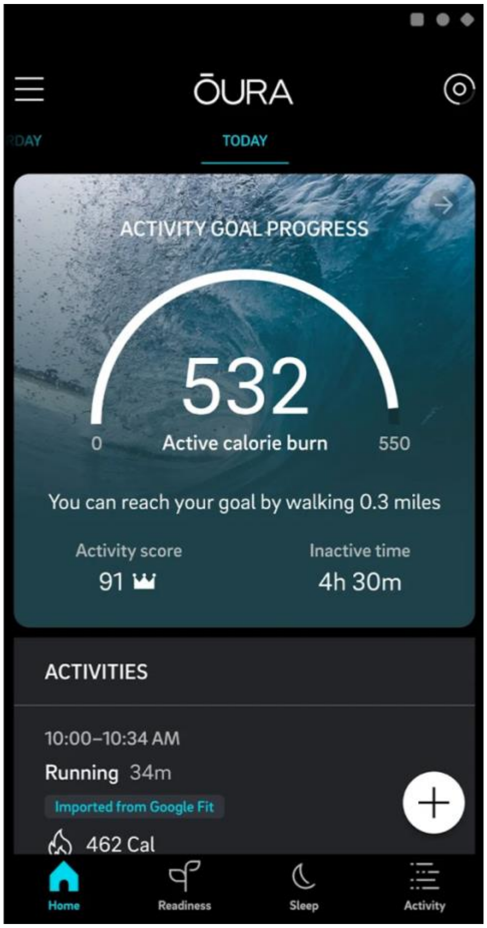	Automatic measuring of activity (steps, distance, active time, calories) and sleep (several metrics).<br><br>Oura ring works only with mobile app and the ring has no interface or indicators.	Daily activity and inactivity count is presented in the mobile app. Goal is adapted to recovery value (readiness score). Presents its own activity score.<br><br>Inactivity alert and activity goal reached feedback is given in mobile app.

<table>
<tr><td>Brand
Name of the mobile application</td><td>Self-monitoring features</td><td>Feedback features</td></tr>
<tr><td>Garmin
Garmin Connect application (iOS, Android)

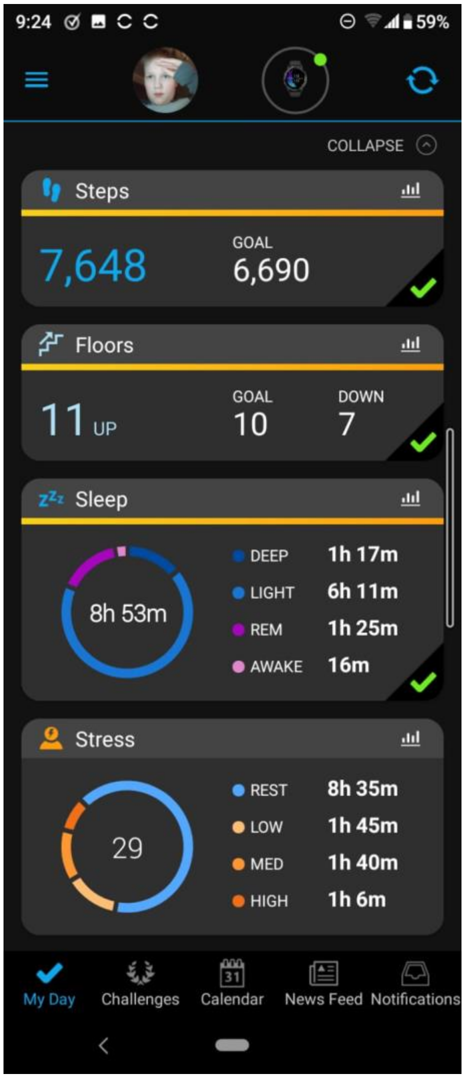
</td><td>Automatic measuring of activity (e.g. steps, distance, floors, active time, calories) and sleep (several metrics).

Presents statistics of all measured issues with different time-frames, e.g. weekly and monthly views.

Several advanced features depending on the sensors available in the wrist device.

Training features record rich data, including heart rate, pace, distance etc. using maps.

Works only with Garmin wrist devices.</td><td>Presents daily activity and several other scores in the main page of the mobile app and in the wrist device.

Enables setting goals and monitoring goal progress (both mobile app and device).

Gives feedback on goal attainment.</td></tr>
</table>

Brand<br>Name of the mobile application	Self-monitoring features	Feedback features
Fitbit<br>Fitbit application (iOS, Android)<br>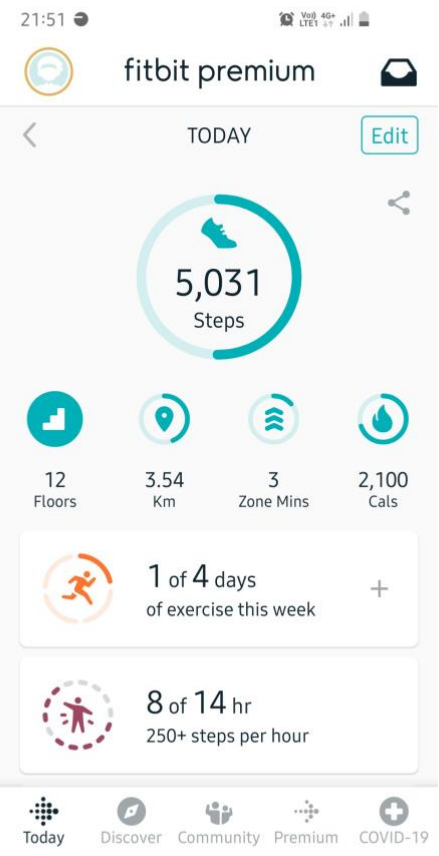 	Automatic measuring of activity (e.g. steps, distance, active time, calories) and sleep (several metrics).<br><br>Presents statistics of all measured issues with different time-frames, e.g. weekly and monthly views.<br><br>Several advanced features depending on the sensors available in the wrist device.<br><br>Training features record rich data, including heart rate, pace, distance etc.<br><br>Works only with Fitbit wrist devices.	Presents daily activity scores and parameters and several other scores in the main page of the mobile app and in the wrist device.<br><br>Enables setting goals and monitoring goal progress (both mobile app and device).<br><br>Gives feedback on goal attainment.

Brand<br>Name of the mobile application	Self-monitoring features	Feedback features
Samsung<br>Samsung Health (iOS, Android)<br>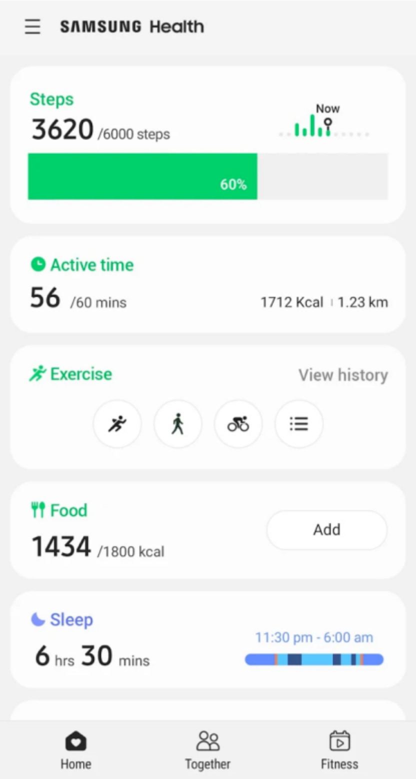	Automatic measuring of activity (steps, exercise, inactivity, calories etc.) and sleep (several metrics). Some features require Galaxy Watch as tracker.<br><br>Presents statistics of activities with different time-frames, e.g. weekly and monthly views.<br><br>Basic sensing data is provided by phone sensors and some features of the app function without trackers.	Activity is presented both in app and in wrist device.<br><br>Both the wrist device and mobile application shows current status of each measured parameter.<br><br>Both app and tracker give feedback on goal attainment.
