# Supplementary material for: Need for Cognition Among Users of Self-Monitoring Systems for Physical Activity: Survey Study
Source: JMIR Form Res. 2021 Oct 14;5(10):e23968. doi: 10.2196/23968 (PMC8554677; doi:10.2196/23968)
Supplement: Multimedia Appendix 3 [file formative_v5i10e23968_app3.pdf]

Multimedia Appendix 3

Cross-loadings of each measurement item.

	Feedback	Perceived credibility	Perceived persuasiveness	Self-monitoring	NFC
cred_1	0.386	**0.842**	0.534	0.472	0.234
cred_2	0.399	**0.835**	0.529	0.461	0.131
cred_3	0.398	**0.822**	0.533	0.465	0.199
cred_4	0.500	**0.803**	0.564	0.563	0.221
cred_5	0.401	**0.709**	0.410	0.379	0.149
dial_1	**0.867**	0.447	0.489	0.591	0.162
dial_2	**0.835**	0.421	0.423	0.538	0.194
dial_3	**0.843**	0.455	0.517	0.574	0.121
nfc_2	0.136	0.244	0.175	0.168	**0.774**
nfc_6	0.168	0.148	0.215	0.126	**0.782**
nfc_7	0.148	0.180	0.249	0.148	**0.844**
nfc_8	0.146	0.193	0.141	0.107	**0.747**
nfc_9	0.125	0.170	0.130	0.094	**0.763**
pepe_1	0.526	0.681	**0.837**	0.607	0.279
pepe_2	0.467	0.499	**0.858**	0.568	0.101
pepe_4	0.489	0.585	**0.877**	0.556	0.221
pepe_5	0.398	0.356	**0.788**	0.457	0.231
prim_1	0.556	0.525	0.613	**0.825**	0.131
prim_2	0.541	0.466	0.531	**0.843**	0.134
prim_4	0.570	0.468	0.481	**0.819**	0.154
